# Election Turnout Statistics in Many Countries: Similarities, Differences, and a Diffusive Field Model for Decision-Making

**DOI:** 10.1371/journal.pone.0036289

**Published:** 2012-05-16

**Authors:** Christian Borghesi, Jean-Claude Raynal, Jean-Philippe Bouchaud

**Affiliations:** 1 Centre d’Analyse et de Mathématique Sociales (CAMS), CNRS/EHESS, Paris, France; 2 EHESS, Division Histoire, Paris, France; 3 Capital Fund Management, Paris, France; Northwestern University, United States of America

## Abstract

We study in details the turnout rate statistics for 77 elections in 11 different countries. We show that the empirical results established in a previous paper for French elections appear to hold much more generally. We find in particular that the spatial correlation of turnout rates decay logarithmically with distance in all cases. This result is quantitatively reproduced by a decision model that assumes that each voter makes his mind as a result of three influence terms: one totally idiosyncratic component, one city-specific term with short-ranged fluctuations in space, and one long-ranged correlated field which propagates diffusively in space. A detailed analysis reveals several interesting features: for example, different countries have different degrees of local heterogeneities and seem to be characterized by a different propensity for individuals to conform to the cultural norm. We furthermore find clear signs of herding (i.e., strongly correlated decisions at the individual level) in some countries, but not in others.

## Introduction

Empirical studies and models of election statistics is a classical field of Political Economy [1]–[5]. This subject has attracted considerable attention in the recent physics literature, see e.g. [6]–[16]. In [17], the present authors have studied the statistical regularities of the electoral turnout rates, based on spatially resolved data from 13 French elections since 1992. Two striking features emerged from our analysis: first, the distribution of the logarithmic turnout rate 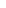 (defined precisely below) was found to be remarkably stable over all elections, up to an election dependent shift. Second, the spatial correlations of 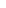 was found to be well approximated by an affine function of the *logarithm* of the distance between two cities. Based on these empirical results, we proposed that the behaviour of individual agents is affected by a space dependent “cultural field”, that encodes a local bias in the decision making process (to vote or not to vote), common to all inhabitants of a given city. The cultural field itself can be decomposed into an idiosyncratic part, with short range correlations, and a slow, long-range part that results from the diffusion of opinions and habits from one city to its close-by neighbours. We showed in particular that this local propagation of cultural biases generates, at equilibrium, the logarithmic decay of spatial correlations that is observed empirically [17].

The aim of the present note is to provide additional support to these rather strong statements, using a much larger set of elections from different countries in the world. We discuss in more depth the approximate universality of the distribution of turnout rates, and show that some systematic effects in fact exist, related in particular, to the size of the cities. We also confirm that the logarithmic decay of the spatial correlations approximately holds for all countries and all elections, with parameters compatible with our diffusive field model. The relative importance of the idiosyncratic, city dependent contribution and of the slow diffusive part is however found to be strongly dependent on countries. We also confirm the universality of the logarithmic turnout rate for different elections, for different regions or for different cities, provided the mean and the width of the distribution is allowed to depend on the city size. Overall, our empirical analysis provides further support to the binary logit model of decision making, with a space dependent mean (the cultural field mentioned above).

## Results and Discussion

### Data and Observables

We have analysed the turnout rate at the scale of municipalities for 77 elections, from 11 different countries. For some countries, the number of different elections is substantial: 22 from France (Fr, 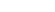 municipalities in mainland France), 13 from Austria (At, 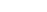 municipalities), 11 from Poland (Pl, 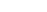 municipalities), 7 from Germany (Ge, 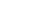 municipalities), while for others we have less samples: 5 from Canada (Ca, 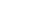 municipalities), 4 from Spain (Sp, 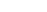 municipalities in mainland Spain), 4 from Italy (It, 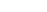 municipalities in mainland Italy), 4 from Romania (Ro, 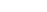 municipalities), 3 from Mexico (Mx, 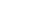 municipalities), 3 from Switzerland (CH, 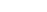 municipalities) and 1 from Czech Republic (Cz, 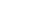 municipalities). More details on the nature of these elections and some specific issues are given in Appendix S1.

For each municipality and each election, the data files give the total number of registered voters 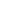 and the number of actual voters 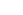, from which one obtains the usual turnout rate 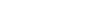. For reasons that will become clear, we will instead consider in the following the logarithmic turnout rate (LTR) 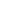, defined as:
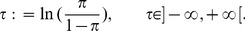
(1)Because we know the geographical location of each city, the knowledge of 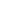 for each city enables us to create a map of the field 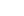 and study its spatial correlations.

### Statistics of the Local Turnout Rate

Whereas the average turnout rate is quite strongly dependent on the election (both on time and on the type of election – local, presidential, referendum, etc.), the distribution of the shifted LTR 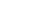 was found to be remarkably similar for the 13 French elections studied in [17]. (The notation 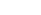 means a flat average over all cities, i.e. not weighted by the population 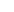 of the city.) The LTR standard-deviation, skewness and kurtosis were found to be very similar between different elections. The distribution 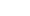 of the shifted and rescaled LTR,
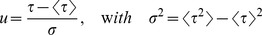
(2)was found to be very close in the Kolmogorov-Smirnov (KS) sense.

**Figure 1:**
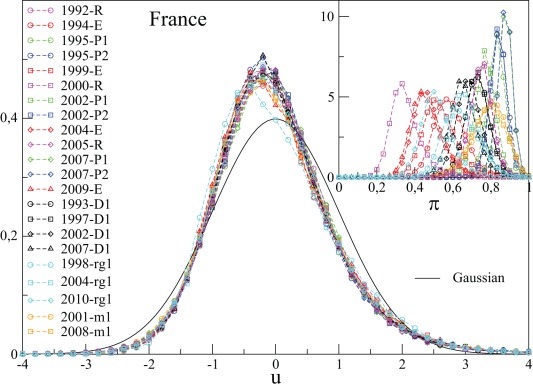
Probability distribution of the rescaled variable 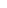 over all *communes* for France. A standardized Gaussian is also shown. The inset similarly shows the probability distribution of the usual turnout rate 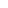. We use the same symbols and color codes for the French elections throughout this paper.

We have extended this analysis to the 9 new election data in France, and to all new countries mentioned above. For France, the *Elections Municipales* (election of the city mayor), not considered in [17], have a distinctly larger standard deviation than national elections. However, 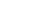 is again found to be similar for all the French elections, except the *Régionales* of 1998 and 2004. These happen to be coupled with other local elections in half municipalities, which clearly introduces a bias. The distributions 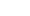 for all elections in France are shown in Fig. 1 and compared to a Gaussian variable. The distribution is clearly non Gaussian, with a positive skewness equal to 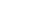 and a kurtosis equal 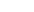. A more precise analysis consists in computing the KS distances between each pair of elections. We recall here that a KS distance of 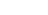 corresponds to a 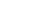 probability that the two tested distribution coincide, while 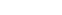 corresponds to a 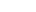 probability. Removing the *Régionales*, we find that the KS distance 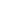 averaged over all pairs of elections is equal to 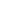, with a standard deviation of 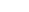. These numbers are slightly too large to ascertain that the distributions are exactly the same since in that case the average 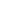 should be equal to 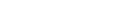. On the other hand, these distances are not large either (as visually clear from Fig. 1), meaning that while systematic differences between elections do exist, they are quite small. We will explain below a possible origin for these differences.

The same analysis can be done for all countries separately; as for France, we find that 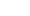 for different elections are all similar, except for Germany for which 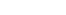 – see Table 1, where we show the mean and the standard-deviation of KS distances between elections of a given country, and of the skewness and kurtosis of the distributions 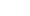 in a given country. Note that the values of 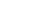 are close to 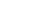 for Italy and Poland. On the other hand, these distributions is clearly found *not* to be identical across different countries. Table 2 shows the matrix of KS distances between countries “super-distributions”. (A “super-distribution” of 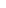 of a country is obtained by aggregating the appropriately shifted LTR distributions over all “compatible” elections. Compatible elections have roughly the same distribution 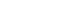, i.e. *without* normalization by its standard-deviation. They are chosen as follows: for Canada and Poland all elections; for France all pure national elections (nor combined with local elections, i.e. all elections apart from *Régionales* in 1998 and 2004, and *Municipales* in 2001 and 2008); for Mexico, Chamber of Deputies in 2003 and 2009; for Germany Chamber of Deputies in 2005 and 2009; all Chamber of Deputies elections for Austria, Spain, Italy and Switzerland; and for Romania, all elections apart from its European Parliament election (see Appendix S1, for more details).) The values of 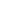 are all large, except for the pairs France-Czech Republic, France-Switzerland, Spain-Switzerland, Spain-Romania and Switzerland-Czech Republic.

**Table 1:** Mean and standard-deviation of KS distances (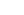) between all pairs of elections within each country.

Country	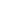	skewness	kurtosis	Country	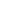	skewness	kurtosis
Austria	1.44 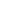 0.54	0.10 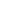 0.38	0.53 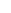 0.81	Canada	1.23 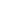 0.39	−0.40 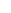 0.39	4.4 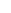 0.9
	(0.93 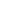 0.19)	(−0.13 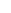 0.21)	(0.54 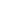 0.43)		(1.23 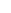 0.39)	(−0.40 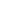 0.39)	(4.4 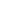 0.9)
France	1.49 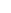 0.47	1.07 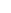 0.15	4.7 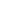 1.4	Germany	3.0 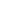 1.1	0.48 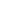 0.30	1.6 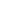 0.9
	(1.42 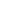 0.45)	(1.10 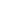 0.14)	(5.1 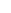 0.9)		(0.81)	(0.20 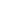 0.05)	(1.53 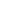 0.04)
Italy	0.70 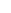 0.09	−0.45 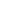 0.11	1.01 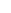 0.02	Mexico	1.28 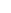 0.35	0.32 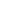 0.09	1.1 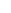 0.8
	(0.68)	(−0.45 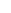 0.15)	(1.01 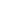 0.003)		(1.19)	(0.35 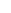 0.11)	(1.6 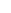 0.3)
Poland	0.80 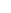 0.20	0.12 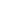 0.26	0.38 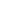 0.42	Romania	1.06 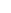 0.39	0.05 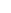 0.43	1.5 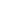 0.4
	(0.80 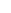 0.20)	(0.12 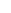 0.26)	(0.38 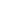 0.42)		(0.95 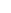 0.36)	(−0.14 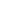 0.25)	(1.6 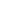 0.4)
Spain	1.78 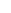 0.68	0.27 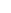 0.25	1.8 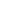 1.1	Switzerland	1.67 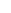 0.43	0.51 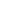 0.08	1.4 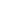 1.4
	(1.24)	(0.07 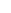 0.21)	(2.5 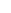 1.2)			(0.47)	(2.9)

Mean and standard-deviation of skewness and kurtosis of distributions of 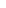 over all municipalities is also given for each country. In parentheses, the same measures but restricted to compatibles elections in each country.

**Table 2:** Kolmogorov-Smirnov distance, 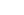, between the “super-distributions” corresponding to different countries.

	Ca	Cz	Fr	Ge	It	Mx	Pl	Ro	Sp	CH
At	*4.60*	*3.24*	*5.01*	**1.49**	1.58	2.31	1.62	1.57	2.43	2.25
Ca		2.45	*6.72*	*7.15*	*4.62*	*4.06*	*6.62*	2.78	*3.53*	**1.44**
Cz			**0.93**	*4.84*	*3.65*	2.12	*3.16*	1.94	1.71	**0.58**
Fr				*8.00*	*6.13*	2.18	*5.61*	2.66	2.28	**0.83**
Ge					1.73	2.81	2.32	2.85	*3.74*	2.83
It						*3.13*	*3.12*	2.05	*3.17*	2.58
Mx							1.83	1.95	2.19	1.87
Pl								1.99	2.41	2.06
Ro									**0.95**	**1.39**
Sp										**1.11**

In italic, normal and bold text, respectively 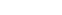, 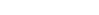 and 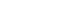.

**Figure 2:**
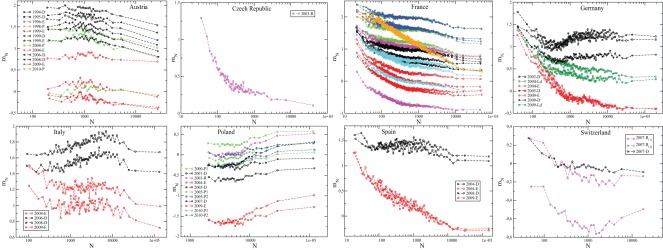
Average value, 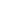, of the conditional distribution 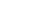. These quantities are obtained as averages over bins with 100 (200 for France) municipalities of size 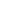. See Fig. S1 in Appendix S1 for Canada, Mexico and Romania.

**Figure 3:**
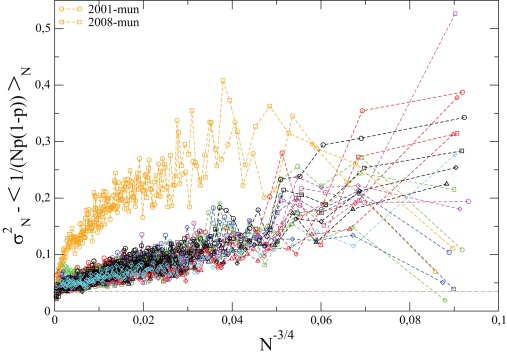
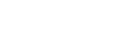
 as a function of 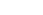 for French elections. Each point comes from around 300 *communes* of size 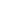. Dashed line: 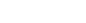 as extracted from the spatial correlations of 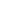 (cf. Tab. 7). The 1998 and 2004 *Régionales* elections are excluded here.

**Figure 4:**
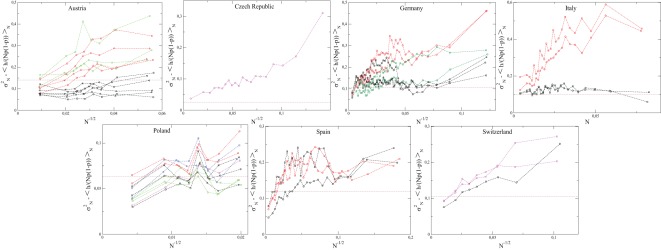
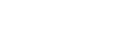
 as a function of 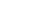 for each election. (The exponent 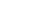 in 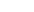 is here equal to 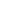 for all countries in order to take into account each country in the same way.) These quantities are obtained as averages over bins with 300 municipalities of size 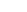. The dashed line corresponds to 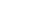 as extracted from the spatial correlations of 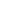 (cf. Fig. 9). For election labels, see Figs. 1, 2. See Fig. S2 in Appendix S1 for Canada, Mexico and Romania.

In order to understand better these results, one should first realize that the statistics of the LTR does in fact strongly depend on the size of the cities. This was already pointed out in [17], [18]. For example, the average LTR for all cities of size 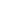 (within a certain interval), that we denote as 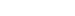, is distinctly 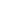 dependent, see Fig. 2 and Fig. S1 in Appendix S1. In most cases, the average turnout rate is large in small cities and declines in larger cities, with notable exceptions: for example, the trend is completely reversed in Poland, with more complicated patterns for parliament elections in Italy or Germany. Similarly, the standard-deviation of 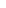, 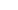, also depends quite strongly on 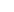 (see below Figs. 3, 4, and Fig. S2 in Appendix S1).

However, the distribution 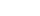 of the rescaled variable 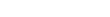 over all cities of size 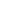 for each election can be considered to be identical from a KS point of view, both within the same country for different 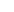 but now also across different countries, at least when 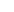 is large enough (arguments will be provided below to understand why this should be expected).

For example, the average KS distance between 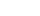 distributions corresponding to different ranges of 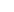 in France is equal to 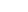, with standard-deviation 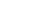. These numbers are respectively 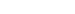, 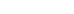 and 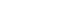 for Italy, Spain and Germany. (We have excluded the smallest cities, 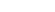, that have a distinctly larger KS distance with other cities – see below. Bins, ranked according to the municipality size 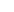 contain each around 500 municipalities.) In Table 3, we show for different bins of 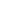 values the mean and standard-deviation KS distance between countries, illustrating that all distributions are statistically compatible, at least when 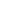 is large enough.

**Table 3:** Mean and standard-deviation over all pairs of countries of the KS distance 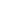 between the aggregated 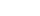 distributions in each country, for different values of 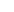.

	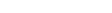	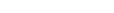	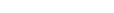	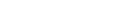	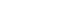
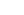	1.47 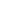 0.77	1.38 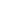 0.65	0.94 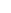 0.48	0.91 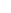 0.46	0.95 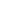 0.48

**Figure 5:**
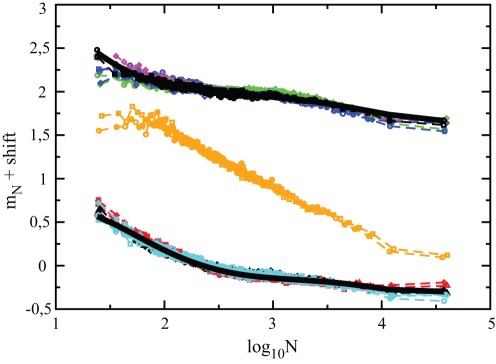
Shifted 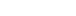 as a function of 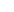 for French elections. Three families of elections clearly appear. a) Top curves: “important” national elections (Presidential, Referendums, Parliament); b) Bottom curves: less important national elections (European, *Régionales*); and c) Middle curves: *Municipales* (see text). Each point comes from the average over around 200 *communes* of size 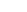.

Now, even if 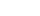 was really universal and equal to 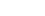, 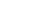 would still reflect the country-specific (and possibly election-specific) shapes of 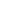 and 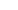, and the country-specific distribution of city sizes, 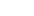. Indeed, one has:
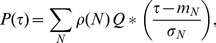
(3)which has no reason whatsoever to be country independent. But since for a given country the dependence on 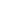 of 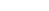 and 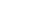 tends to change only weakly in time, the approximate universality of 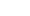 for a given country follows from that of 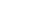. In fact, French national elections can be grouped into two families, such that the dependence of 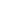 on 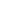 is the same within each family but markedly different for the two families (see next section and Fig. 5 below). Restricting the KS tests to pairs within each families now leads to an average KS distance for 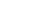 of 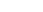 with a standard deviation 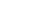 (identical for the two families), substantially smaller than 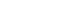 from Table 1. This goes to show that the election specific shape of 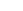 is indeed partly responsible for the weak non-universality of 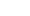 within a given country.

Zooming in now on details, we give in Table 4 the KS distance between 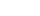 aggregated over all elections of a country and a normalized Gaussian, for different ranges of 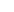 and different countries. The skewness and kurtosis of the distribution 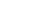 and the KS distance to a Gaussian, aggregated over all 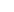, are given in Table 5 for different countries, and aggregated over countries for fixed 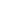 in Table 6. Two features emerge from these Tables:

- While for some countries (Czech Republic, Spain and Mexico) the deviation of 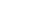 from a Gaussian appear small (both measured by KS or by the skewness and kurtosis), such an assumption is clearly unacceptable for Italy and Germany, for which the KS distance is large for all 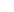 (see Table 4) and a substantial negative skewness can be measured. Furthermore, the aggregated distribution (over all 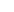) is clearly incompatible with a Gaussian except in the Czech Republic, Spain and Mexico – see Table 5.
- Table 6 shows an interesting systematic 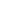 dependence of the distance to a Gaussian, which is on average smaller for larger 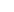
s, and maximum for small cities. This suggests that although the KS tests is unable to distinguish strongly the 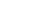 for different 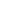, there is in fact a systematic evolution for which we provide an argument below. In fact, as clearly seen in Table 3, the average KS distance between the 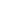 of different countries is also systematically smaller as 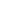 increases.

**Table 4:** KS distance between 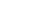 and a normalized Gaussian for different ranges of 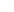 and for different countries.

Country	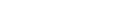	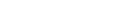	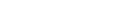	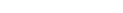	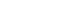
Austria	2.03	1.82	0.76	0.98	1.58
Canada	3.48	1.09	0.60	0.53	0.59
Czech Rep.	0.63	0.73	0.55	0.37	0.61
France	2.50	2.15	1.18	0.71	0.86
Germany	1.75	2.78	2.55	2.49	3.08
Italy	2.69	3.74	3.11	2.32	0.88
Mexico	1.50	0.79	0.55	0.97	0.48
Poland	0.45	1.45	0.89	1.40	1.20
Romania	1.73	1.48	1.14	0.63	0.92
Spain	0.70	0.83	0.71	0.63	0.69
Switzerland	1.38	1.49	0.65	0.69	0.44

**Table 5:** KS distance (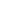) to a standardized Gaussian, and low-moment skewness (skew) and kurtosis (kurt) of aggregated distributions 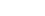.

Country	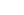	skew	kurt
Austria	2.63	−0.05	0.15
Canada	2.93	−0.75	2.14
Czech Rep.	0.83	−0.32	0.30
France	2.55	−0.02	0.31
Germany	4.09	−0.21	0.05
Italy	5.61	−0.67	0.79
Mexico	1.21	0.12	−0.06
Poland	2.13	0.18	0.58
Romania	2.36	−0.06	1.25
Spain	1.03	−0.16	0.41
Switzerland	1.85	0.24	0.88

Data are aggregated over all 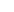 for each country.

**Table 6:** KS distance (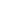) to a standardized Gaussian, and low-moment skewness (skew) and kurtosis (kurt) of aggregated distributions 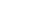. Data are aggregated over all countries for fixed 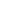.

Range of 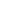	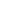	skew	kurt
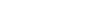	2.25	−0.07	0.43
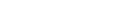	3.50	−0.12	0.44
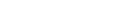	2.90	−0.12	0.42
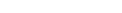	1.74	−0.13	0.31
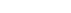	1.74	−0.19	0.43

### A Theoretical Canvas

In order to delve deeper into the meaning of the above results, we need a theoretical framework. In [17], we proposed to extend the classical theory of choice to account for spatial heterogeneities. A registered voter 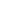 makes the decision to vote (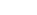) or not (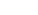) on a given election. We can view this binary decision as resulting from a continuous and unbounded variable 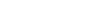 that we called *intention* (or propensity to vote). The final decision depends on the comparison between 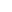 and a *threshold value* 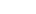: 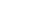 when 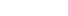, and 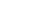 otherwise. In [17], the intention 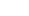 of an agent at time 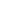 who lives in a city 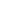, located in the vicinity of 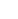, was decomposed as:
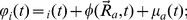
(4)where 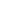 is the instantaneous and idiosyncratic contribution to the intention that is specific to voter 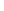, and 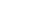 and 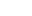 are fields that locally bias the decision of agents living in the same area. The first field 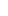 is assumed to be smooth (i.e. slowly varying in time and space), as the result of the local influences of the surroundings. This is what we called a “cultural field”, that transports (in space) and keeps the memory (in time) of the collective intentions. The second field 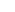, on the other hand, is city- and election-specific, and by assumption has small inter-city correlations. It reflects all the elements in the intention that depend on the city: its size, the personality of its mayor, the specific importance of the election that might depend on the socio-economic background of its inhabitants, as well as the fraction of them who recently settled in the city, etc. (See [17] for a more thorough discussion of Eq. (4).)

Consider now 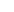 agents living in the same city, i.e. with under the influence of same field values 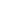 and 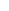. The turnout rate 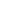 is by definition:
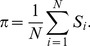
(5)For 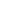 sufficiently large, and if the agents make *independent decisions*, the Central Limit Theorem tells us that:
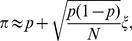
(6)where 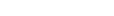 is the probability that the conviction of the voter is strong enough, and 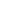 is a standardized Gaussian noise. If, on the other hand, agents make correlated decisions (for example, everybody in a family decides to vote or not to vote under the influence of a strong leader), one expects the variance of the noise term to increase by a certain “herding” factor 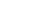, which measures the average size of strongly correlated groups. Therefore we will write more generally:



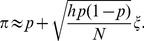
(7)Following a standard assumption in Choice Theory [19], we take the idiosyncratic 
’s to have a logistic distribution with zero mean and standard-deviation 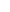, in which case the expression of 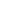 becomes:
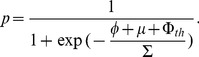
(8)This allows one to obtain a very simple expression for the LTR 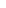:
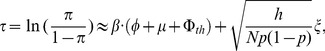
(9)where 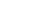. Therefore, in this model, the statistics of 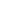 directly reflects that of the cultural and idiosyncratic fields.

Let us work out some consequences of the above decomposition, and how they relate to the above empirical findings. Since the cultural field 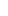 is by definition not attached to a particular city, it is reasonable to assume that 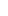 and 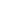 are uncorrelated. Without loss of generality, one can furthermore set 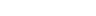. Therefore:
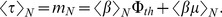
(10)Two extreme scenarios can explain the 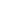 dependence of 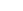: one is that the dispersion term 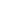 is strongly 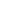 dependent while the statistics of 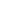 is 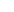 independent, the other is that 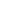 is essentially constant and reflects an intrinsic dispersion common to all voters in a population, while the average of the city-dependent field 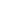 depends strongly on the size of the city. Of course, all intermediate scenarios are in principle possible too, but the data is not precise enough to hone in the precise relative contribution of the two effects. Here, we want to argue that the dependence of 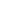 on 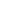 is likely to be dominant. Indeed, if the first scenario was correct, one should observe:
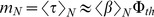
(11)The decrease of 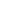 as a function of 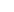 would therefore mean that 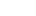 itself is a decreasing function of 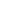 when the mean LTR is positive. This is a priori reasonable: one expects more heterogeneity (and therefore a larger 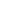, and a smaller 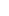) in large cities than in small cities. However, the same model would imply a smaller dependence on 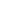 for low turnout rates, and even an inverted dependence of 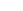 on 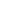 for elections with a very low turnover rate, such that 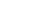. This is not observed: quite on the contrary, the 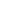 dependence is compatible with a mere vertical shift for similar elections, see Fig. 5.

On the other hand, a model where 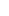 is constant, independent of 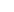 and to a first approximation on the election, leads to:
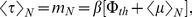
(12)which appears to be a good representation of reality. The dependence of 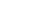 – the average propensity to vote – on 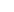, could be the result of several intuitive mechanisms: for example, voters in small cities are less likely to be absent on election day (usually a sunday in France); the result of an election is sometimes more important in small cities than in large cities (for example, election of the mayor); the social pressure from the rest of the community is stronger in small cities; all these effects suggest that the average turnout rate is stronger in small cities. In order to explain the opposite behaviour (as in Poland), or a non-monotonous dependence, as in Italy or Germany for parliament elections, a systematic dependence of 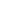 on 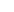 might be relevant, although one should probably dwell into local idiosyncracies.

Figure 5 suggests that in France three families of elections clearly appear: a) “important” national elections (Presidential, Referendums, Parliament), for which 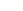 shows a change of concavity around 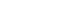; b) less important national elections (European, *Régionales*) for which the average turnout is low, for which the change of concavity is absent; and c) *Municipales* for which the variation of 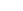 between small and large cities is the largest (as can be expected a priori). Note that the difference 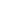 between the mean LTR for small and large cities is markedly different in the three cases: 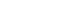 in case a), 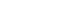 in case b), and 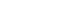 in case c).

As a first approximation, we thus take 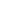 to be constant for all cities. The standard-deviation of 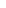 over all cities of a given size then writes:
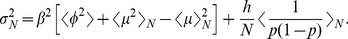
(13)We show in Fig. 3 the quantity 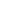 minus the trivial binomial contribution, i.e. the last term of the right hand side of the above equation, as a function of 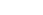, for French elections. The exponent 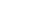 results from the best fit of 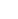 (minus the trivial binomial contribution) as a power law of 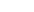. As predicted by the above model, we see that the 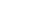 limit is clearly positive 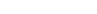, and to a good approximation independent of the election – including the *Municipales*: although the 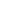 dependence of 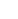 is found to be markedly different (as 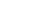), this quantity still extrapolates to the same asymptotic value. If one believes that our interpretation of 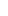 as a persistent cultural field is correct, there is in fact no reason to expect that 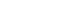 should change at all from election to election. The above result is therefore compatible with the fact that 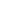 is to a first approximation election independent, as already suggested by Fig. 5 above. The same results hold for all other countries, although the statistics is not as good as in the case of France: the asymptotic value of 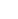 for 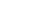 is only weakly dependent on the election, and 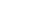 in the range 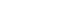 for all countries. Furthermore, the 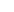-dependence of 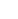 is found to be roughly compatible with 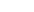 with 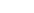 in all cases. We choose to plot the results as a function of 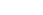 for all these other countries, as to suggest that 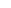 is in some cases larger than 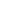 (like for Cz), or less than 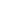 (like for Ge).

If 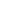 is constant, the 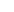-dependent contribution of 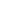 must come from the variance of the city-specific contribution 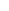. A simple-minded model for the statistics of 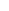 predicts a variance that should decrease as 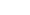. Indeed, a large city can be thought of as a patchwork of 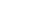 independent small neighbourhoods, each with a specific value of 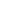. The effective value of 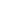 for the whole city has a variance that is easily found to be reduced by a factor 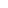, and therefore 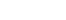. A weaker dependence of 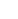 on 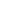 signals the existence of strong inter-neighbourhood correlations (or strong heterogeneities in the size of neighbourhoods), that lead to a reduction of the effective number of independent neighbourhood from 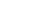 to 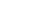 with 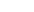 (see [20], [21] for a related discussion). These inter-neighbourhood correlations are indeed expected, since some of the socio-economic and cultural factors affecting the decision of voters are clearly associated to the whole city. Interestingly, these correlations should be stronger for local elections, which is indeed confirmed by the fact that 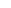 is markedly smaller for the *Municipales* elections in France. We therefore find the interpretation of the anomalous 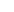 dependence of 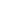 as due to the city-specific contribution 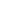 rather compelling.

Let us now turn to the distribution of the rescaled variable 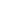. Within the above model, and again assuming that 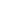 is constant, one finds that:
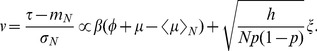
(14)


The last “binomial” term quickly becomes Gaussian as 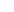 increases, and is at least four times smaller than the first two terms when 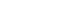 (when 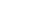). Since the cultural field 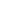 is, according to the model proposed in [17], the result of averaging random influences over long time scales and large length scales, one expects, from the Central Limit Theorem, that 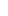 is close to a Gaussian field as well. However, the statistics of 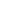 has no reason to be Gaussian for small cities 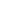, for which it reflects local and instantaneous idiosyncracies, and for which no averaging argument can be invoked. The “universality” of 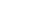 across countries is therefore probably only apparent, since there is no reason to expect that the distribution of 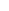 is independent of the country. In fact, 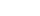 in countries like Italy, Germany and the Czech Republic do exhibit a stronger skewness than in other countries. Still, according to the above discussion, the contribution of different neighbourhoods to 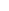 must average out as 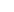 increases, and one expects the distribution of 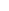 itself to become more and more Gaussian as 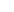 increases.

To sum up: the random variable 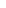 is the sum of three independent random variables, two of which can be considered as Gaussian, while the third has a distribution that depends on 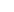 and becomes more Gaussian for large 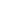, with a variance that decreases as 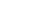. This allows one to rationalize the above empirical findings on the distributions 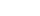: these are more and more Gaussian as 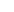 increases, and closer to one another for different countries, since the country specific contribution 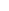 becomes smaller (as 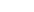) and itself more Gaussian.

It is instructive to compare the relative contribution to the variance of the turnout rates of the cultural field 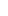 on the one hand, and of the city-specific field on the other. The latter can be obtained by subtracting from the total variance of the LTR, 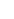, the contribution of the cultural field 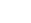 which is obtained as the extrapolation of 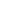 to 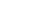 (see Figs. 3 and 4) and the average contribution of the binomial noise, 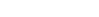. The herding factor 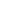 can be estimated using the method introduced in [17], which compares different elections for which the binomial noises are by definition uncorrelated (see Eq. (10) of Ref. [17]). The ratio of 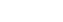 can be seen as an objective measure of the heterogeneity of behaviour in country, i.e. how strongly local idiosyncracies can depart from the global trend. Table 7 gives the ratio 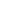 for all studied countries. Using this measure, we find that the most heterogeneous countries are Canada and the Czech Republic (although the ratios for Ca, Mx, Cz and Ge might be overestimated because the data did not allow us to estimate the herding ratio 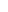 in these two cases) and the most homogeneous ones are Austria, Switzerland and Romania. Not surprisingly, however, the largest value of 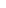 is found for the French *Municipales*, i.e. local elections, for which idiosyncratic effects are indeed expected to be large. Note also that the herding ratio is anomalously high for Romania (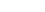), and quite substantial for Poland (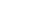). Finally, it is interesting to notice that the quantity 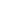 depends only weakly on the country (it varies by a factor 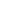 between France and Italy). Since the total intention 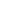 is only defined up to an arbitrary scale, one can always set 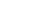. Therefore, we find that the idiosyncratic dispersion 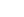 (or the propensity not to conform to the norm encoded by the cultural field) is strongest in France, Poland and the Czech Republic, and weakest in Italy and Austria.

**Table 7:** Decomposition of the total LTR variance into a cultural field component 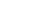, and city-specific component 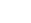, and a binomial component, 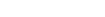, corrected by a herding coefficient 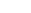.

Country	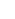	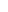	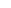	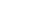 ( 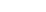 )	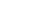 (Eq. 19)	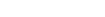	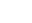	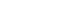
Austria	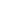	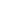	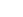	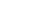	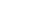	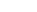	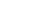	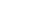
Canada	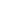	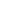	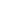	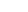	NA	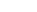	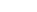	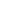
Czech Rep.	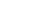	NA 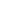	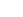	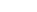	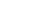	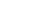	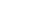	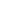
France	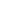	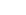	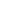	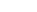	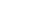	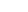	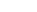	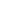
France (mun)	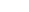	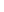	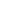	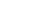	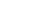	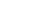	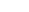	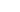
Germany	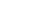	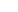	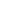	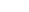	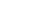	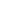	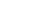	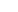
Italy	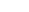	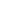	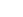	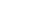	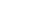	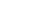	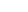	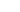
Mexico	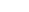	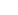	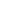	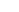	NA	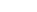	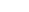	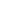
Poland	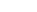	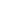	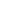	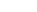	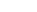	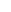	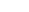	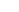
Romania	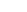	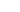	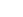	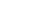	NA	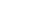	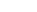	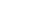
Spain	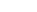	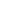	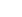	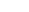	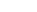	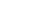	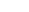	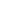
Switzerland	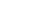	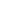	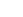	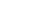	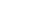	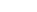	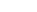	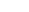

This last term is determined using the method proposed in [17], which leads to a herding coefficient 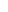 given in the second column. 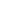: when the direct fit gives a value of 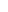 less than unity, we enforce 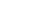. 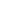: the case of Germany seems to be special, maybe due to a large fraction of postal votes. 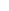: the method to determine 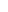 requires more than one election, and therefore cannot be applied to the Czech Republic. In this case, we also set 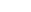 by default. 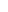: Missing data prevents us from determining 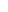 precisely, so we again set 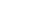 by default. The value of the exponent 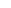 is only indicative, since in some countries the power-law assumption is not warranted, see Fig. 4. We give two values for 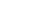: one as the asymptotic extrapolation of 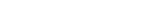 for 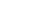 and the second from the rescaling coefficient 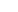, see below and Fig. 9. Both these determinations are only precise to within roughly 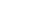.

### Spatial Correlations of Turnout Rates

**Figure 6:**
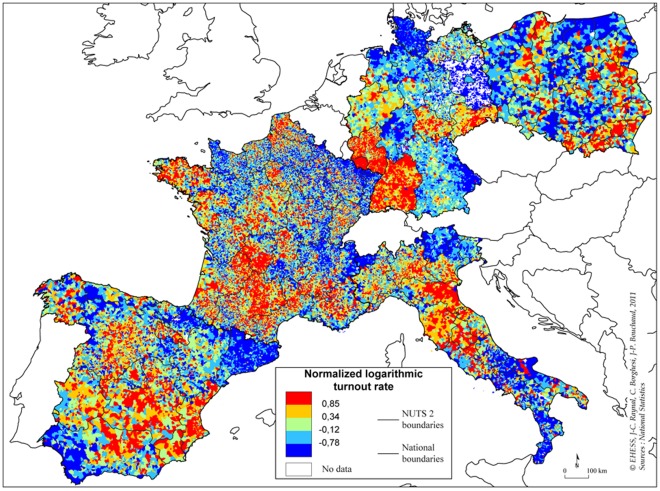
Heat map of the normalized logarithmic turnout rate 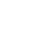, for the 2004 European Parliament election in France, Germany, Italy, Poland and Spain. Germany had nomenclature reform of their municipalities which make more difficult to efficiently join spatial data to electoral data. Note the strongly heterogenous, but long-range correlated nature of the pattern. Note also some strong regionalities, for example in the German regions of Sarre or Bade-Wurtemberg, where the average turnout rate is strong and sharply falls across the region boundaries. In these cases, the implicit assumption of a translation invariant statistical pattern that we make to compute 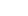 is probably not warranted, and it would in fact be better to treat these regions independently.

**Figure 7:**
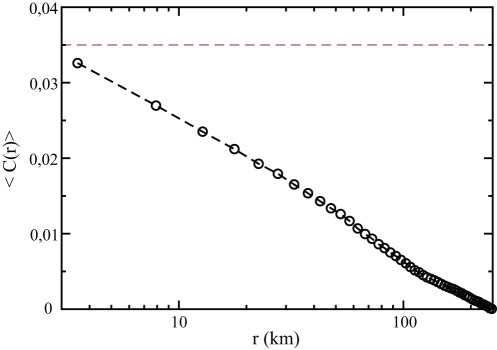
Average of spatial correlations 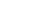 for all French elections (absent the 2 *Municipales* elections). In dashed lines: 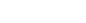, as extracted from the asymptotic (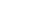) dependence of 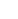.

**Figure 8:**
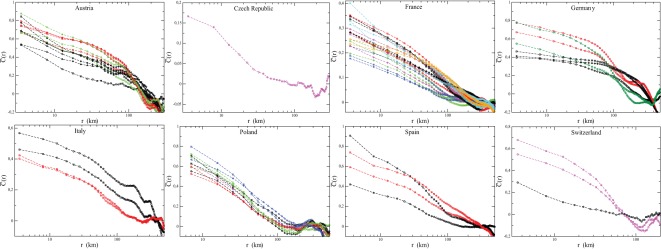
Normalized spatial correlations 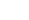 of 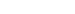 for all countries for which the geographic position of cities is available. The correlation is normalized by the variance of 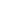, such that 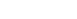. For labels of elections, see Figs. 1, 2.

Another striking empirical finding reported in [17], [18] is the logarithmic dependence of the spatial correlation of the LTR as a function of distance. The spatial pattern of the local fluctuations of the LTR in European countries are shown in Fig. 6. One clearly sees the presence of long-ranged correlations. More precisely, for the 13 French elections studied there, one finds that the spatial correlation of 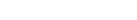 (where 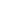 is the spatial location of the city and 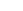 is the average of 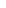 over cities of similar sizes) decreases as:
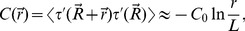
(15)where 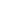 is of the order of the size of the country. We show in Fig. 7 the average 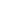 for all French elections (except the two *Municipales* elections) and in Fig. 8 the normalized correlation functions for all elections, separately for each country for which the geographic position of cities is available to us.

Using the above decomposition, and noting that by assumption the fluctuations of 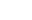 around the suitable size dependent average 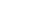 have *short-ranged correlations*, one concludes that the long-range, logarithmic correlations above must come from those of the cultural field 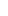. One indeed finds:
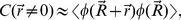
(16)since the other two terms only contribute for 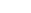. As a consistency check of this decomposition, one should find that 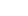 should quickly decay from 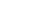 to 
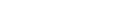
 (e.g. 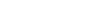 for France). This is indeed seen to be well borne out, see Fig. 7. The agreement between two completely different determination of 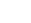 (one using the extrapolation of 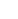 to infinite sizes, and the second using 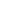) holds very well for France, Italy and the Czech Republic, and only approximately for other countries (see Tab. 7 and Fig. 4).

Inspired by a well-known model in statistical physics where these logarithmic correlations appear, we postulated in [17] that the field 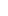 evolves according to a diffusion equation, driven by a random noise, which is meant to describe the exchange of ideas and opinions between nearby cities and the random nature of the shocks that may affect the cultural substrate. As we argued in [17], the fact that people move around and carry with them some components of the local cultural specificity leads to a local propagation of 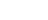. Through human interactions, the cultural differences between nearby cities tend to narrow according to:
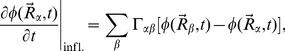
(17)where 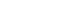 is a symmetric influence matrix, that we assume to decrease over a distance corresponding to regular displacements of individuals, say 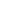 km or so. For concreteness, we take: 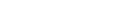. As is well known, the continuum limit of the right hand side of Eq. (17) reads 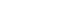, where 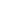 is the Laplacian and 
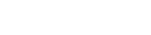
 is a measure of the speed at which the cultural field diffuses. Random cultural “shocks” add to the above equation a noise term 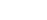.

**Figure 9:**
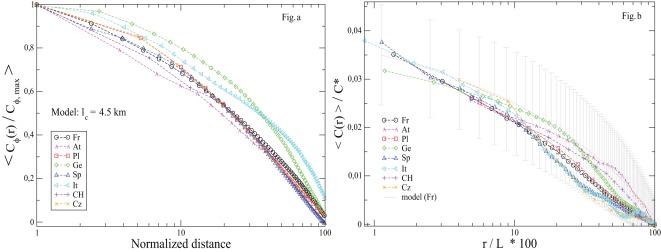
Average of spatial correlation, rescaled. Left: Average over numerical simulations of the model (with 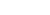 km) with the true positions of all cities for each country. Right: Average over real election data for each country. We also shown the average and standard deviation (coming from different realizations of the noise history 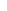, and plotted as error bars) corresponding to the numerical model for French cities.

If cities were located on the nodes of a regular lattice of linear size 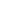, it would be easy to compute analytically the stationary correlation function of the field 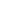. It is found to be given by a logarithm function of distance, provided 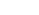:
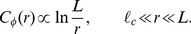
(18)However, the spatial distribution of cities in real countries is quite strongly heterogeneous, which leads to significant deviation from a pure logarithmic decay. In order to compare quantitatively our model with empirical data, we have therefore simulated the model using Eq. (17) with the *exact* locations of all cities for the different countries under consideration. The results, averaged over many histories of the noise term, are shown in Fig. 9-left for 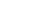 km, (but changing 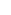 from 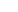 km to 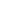 km hardly changes the curves). Quite remarkably, we see that 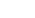 exhibits a significant concavity, very similar to what is observed for the empirical correlations. In order to see that the model is indeed compatible with observations, we have plotted in Fig. 9-right the empirical data superimposed with the prediction of the model for the French case (for which the data is best). The empirical correlation 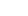 is rescaled by a country dependent value 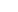 in order to achieve the best rescaling. This value of 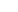 allows us to obtain a second determination of 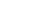, through the relation:
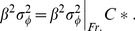
(19)Note however that the numerical model predicts a rather large dispersion around the average result, that comes from a strong dependence on the noise realisation 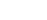. One should therefore expect that the empirical data (which corresponds to only a few histories) departs from the average theoretical curve, in a way perfectly compatible with Fig. 9-right. This also means that there is quite a bit of leeway in determination of 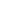, which is only determined to within 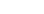. Finally, note that the shape of 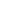 for Germany is significantly different, with a pronounced change of regime around 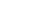 km. This is clearly related to the strong regional idiosyncracies that we discussed in Fig. 6.

We conclude that our numerical model reproduces very satisfactorily the observations for *all studied countries* (with the possible exception of Germany, for the reason noted above). This lends strong support to the existence, conjectured in [17], of an underlying diffusive cultural field responsible for both the long-range correlation (in space) and persistence (in time) of voting habits.

### Conclusion

In this paper, we have shown that the empirical results for the statistics of turnout rates established in [17] for some French elections appear to hold much more generally. We believe that the most striking result is the logarithmic dependence of the spatial correlations of these turnout rates. This result is quantitatively reproduced by a decision model that assumes that each voter makes his mind as a result of three influence terms: one totally idiosyncratic component, one city-specific term with short-ranged fluctuations in space, and one long-ranged correlated field which propagates diffusively in space. The sum of these three contributions is what we call the “intention”. A detailed analysis of our data sets has revealed several interesting (and sometimes unexpected) features: a) the city-specific term has a variance that depends on the size 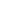 of the city as 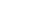 with 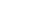, suggesting strong inter-city correlations; b) different countries have different degrees of local heterogeneities, defined as the ratio of the variance of the city-dependent term over the variance of the cultural field; c) different countries seem to be characterized by a different propensity for individuals to conform to a cultural norm; d) there are clear signs of herding (i.e. strongly correlated decisions at the individual level) in some countries, but not in others; e) the statistics of the logarithmic turnout rates become more and more Gaussian as 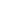 increases.

Although we have confirmed the existence of a diffusive cultural field using election data from different countries, we feel that more work should be done to establish the general relevance of this idea to other decision making processes. It would be extremely interesting to find other data sets that would enable one to study the spatial correlations of decision making. An obvious candidate would be consumer habits – for example the consumption pattern of some generic goods, or the success of some movie, etc.

Finally, we believe that our detailed analysis of the statistics of turnout rates (or more generally of election results) reveals both stable patterns and subtle features, that could be used to test for possible data manipulation or frauds, or to define interesting “democracy” indexes. In that respect, the existence of strong herding effects in some countries is somewhat disturbing.

## Materials and Methods

Appendix S1, gives more information about the set of (public) electoral data studied in this paper. Most of them can be directly downloaded from official websites (see References in Appendix S1). Part of the database used in this paper can also be directly downloaded from [22].

Average values and standard-deviations do not take into account extreme values in order to remove some electoral errors, etc. Electoral values greater than *5 sigma* are not taken into account. For instance let 100 municipalities of size 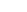 (as in Fig. 2), each one has a LTR 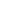 (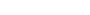). First, 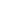 and 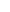 are the average value and the standard-deviation of 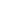 over these 100 municipalities. Next, the final average value 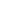 and the final standard-deviation, 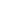, over this sample of 100 municipalities are uniquely evaluated for municipalities, 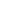, such that 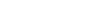.

## Supporting Information

Appendix S1 Details on the data sources and more figures.
(PDF)
