# Supplementary material for: Election Turnout Statistics in Many Countries: Similarities, Differences, and a Diffusive Field Model for Decision-Making
Source: PLoS One. 2012 May 16;7(5):e36289. doi: 10.1371/journal.pone.0036289 (PMC3354000; doi:10.1371/journal.pone.0036289)
Supplement: Appendix S1 — Details on the data sources and more figures. (PDF) [file pone.0036289.s001.pdf]

Election turnout statistics in many countries: similarities, differences, and a diffusive field model for decision-making

Christian Borghesi, Jean-Claude-Raynal and Jean-Philippe Bouchaud

SUPPORTING INFORMATION

Appendix S1: Details on the data sources and more figures

Table S1 shows the nature of the 77 national elections from 11 countries, studied at the municipality scale. Countries are: Austria (At)[1, 2][1], Canada (Ca)[3], Czech Republic (Cz)[4], France (Fr)[5][2], Germany (Ge)[6][3], Italy (It)[7], Mexico (Mx)[8, 9], Poland (Pl)[10–19], Romania (Ro)[20, 21][4], Spain (Sp)[22] and, Swiss (CH)[23][5]. Note that all the studied elections occurred in a same time over all the country (apart from 2 *Länder* elections in Germany) and are free of compulsory voting. Lastly, in our database for Germany, postal votes (*Briehwahlen*) are taken into account in some *Länder*, not in others, which artificially increases turnout heterogeneity between German regions.

Country	n_{el}	n_{mun}	spa	elections
Austria	13	2400	Y	1994-D, 1995-D, 1996-E, 1998-P, 1999-E, 1999-D, 2002-D, 2004-P, 2004-E, 2006-D, 2008-D 2009-E, 2010-P
Canada	5	7700	N	1997-D, 2000-D, 2004-D, 2006-D, 2008-D
Czech Rep.	1	6200	Y	2003-R
France	22	36000	Y	1992-R, 1993-D, 1994-E, 1995-P1, 1995-P2, 1997-D, 1998-rg, 1999-E, 2000-R, 2001-mun, 2002-P1 2002-P2, 2002-D, 2004-rg, 2004-E, 2005-R, 2007-P1, 2007-P2 2007-D, 2008-mun, 2009-E, 2010-rg
Germany	7	12000	Y	2002-D, 2004-Ld, 2005-D, 2009-E, 2009-D, 2010-Ld
Italy	4	7200	Y	2004-E, 2006-D, 2008-D, 2009-E
Mexico	3	2400	N	2003-D, 2006-D, 2009-D
Poland	11	2500	Y	2000-P1, 2001-D, 2003-R, 2004-E, 2005-D, 2005-P1, 2005-P2, 2007-D, 2009-E, 2010-P1, 2010-P2
Romania	4	3200	N	2009-E, 2009-R, 2009-P1, 2009-P2
Spain	4	8000	Y	2004-D, 2004-E, 2008-D, 2009-E
Switzerland	3	2700	Y	2007-$R_{(1)}$, 2007-$R_{(2)}$, 2007-D

TABLE S1. **Nature of elections studied in this paper.** For each country, the number of elections (n_{el}) and the number of municipalities(n_{mun}) in the mainland are written. "Y" (or reversely "N") mentions that municipalities are spatially (spa) localized. For each country, an election is identified by its year date and its nature. D: Chamber of Deputies election; E: European parliament election; P: presidential election (according to the constitution of the country, in only one round); P1 and P2: first and second round of a Presidential election; R: Referendum; Ld: German *Länder* elections; rg: French *Régionales* elections; mun: French *municipales.* For each country elections are given in a chronological order (but the 2009 Romanian Presidential (P) and Referendum (R) elections occurred the same day). Even if an election needs two rounds, only the first one is considered (e.g. the French Chamber of Deputies (D), *Régionales* (rg) and *municipales* (mun) elections) unless the contrary is indicated (e.g. P1 and P2).

Moreover Election turnout statistics have been located, identified and geocoded, based on a set of points, which were obtained by calculating the gravity center of each municipality or the position of the town-hall, and then adding the X and Y coordinates for each of these features. In addition to these coordinates, the objects are described with several attributes: logarithmic turnout rate, τ, normalized logarithmic turnout rate, v, etc. This concerns 8 countries amongst the 11 previous ones [6]: Austria [24], Czech Republic [25], France [26, 27], Germany [28], Italy [29], Poland [30], Spain [31] and Switzerland [32]. This study is limited to mainland municipalities (and each considered country have more than two thousands municipalities). *Lambert 2 étendu* is used for France, while *WGS 84* coordinate system is used for other countries.

Websites given in the References were accessed in December 2011. Part of the database used in this paper can also be directly downloaded from [33].

---

[1] Postal votes (*Wahlkarten*) are not taking account in this paper.

[2] 1998 and 2004 *Régionales* elections occurred at the same time as strictly local elections (*cantonales*, i.e. at a kind of county level) in half of municipalities.

[3] *Land* Parliament elections at time less or equal to 2004 (or 2010) in each *Land* are written here as '2004-Ld' (or '2010-Ld').

[4] The referendum studied here (about the *Parlament unicameral* and the reduction of the maximum of deputies) occurred at the same time than the first round of the Presidential election. Some Romanian electors, not registered in the *lista electorala permanenta*, are able to vote. For this country, we pursue to write N the Number of Register Voters, N_+ the registered electors who take part to the election.

[5] The referendums or *votations* ($R_{(a)}$ and $R_{(b)}$) respectively occurred on March 11th and June 17th 2007.

[6] The Mexican spatial repartition of municipalities is so widely heterogeneous than the spatial study made for other countries is no longer efficient here.

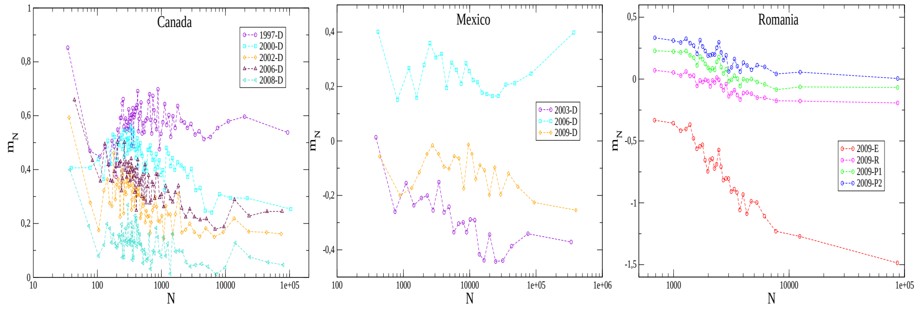

FIG. S1. Average value, m_N, of the conditional distribution $P(\tau|N)$ (as in Fig. 2), for all elections in Canada, Mexico and Romania.

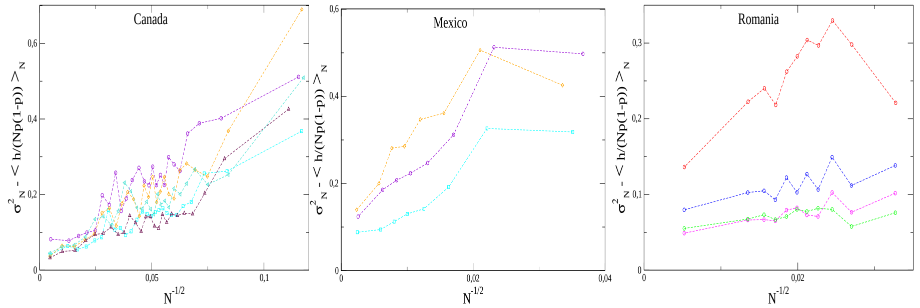

FIG. S2. $\sigma_N^2 - \langle \frac{h}{Np(1-p)} \rangle_N$ as a function of $N^{-1/2}$ for each election (as in Fig. 4), for elections in Canada, Mexico and Romania.

---

[1] http://www.bmi.gv.at/cms/BMI_wahlen/
[2] http://sunsite.univie.ac.at/Austria/elections/
[3] http://www.elections.ca/content.aspx?section=ele&dir=pas&document=index&lang=f
[4] http://www.czso.cz
[5] http://www.interieur.gouv.fr/sections/a_votre_service/elections/resultats
[6] *Amt für Statistik Berlin-Brandenburg*, DVD: Statistik lokal, Ausgabe 2004, 2006, 2010
[7] http://www.interno.it
[8] http://www.ife.org.mx/documentos/RESELEC/SICEEF/principal.html
[9] http://www.ife.org.mx/portal/site/ifev2/Estadisticas_y_Resultados_Electorales/
[10] http://www.pkw.gov.pl/wybory2000/gminy/index.html
[11] http://www.pkw.gov.pl/katalog/artykul/17706.html
[12] http://referendum.pkw.gov.pl/arkusze/index.html
[13] http://www.pe2004.pkw.gov.pl/
[14] http://www.pkw.gov.pl/pkw2/index.jsp?place=Menu01&news_cat_id=1818&layout=1
[15] http://www.pkw.gov.pl/pkw2/index.jsp?place=Menu01&news_cat_id=1819&layout=1
[16] http://wybory2007.pkw.gov.pl/SJM/EN/WYN/W/index.htm
[17] http://pe2009.pkw.gov.pl/PUE/EN/WYN/W/index.htm
[18] http://prezydent2010.pkw.gov.pl/PZT1/EN/WYN/W/index.htm
[19] http://prezydent2010.pkw.gov.pl/PZT/EN/WYN/W/index.htm
[20] http://www.bec2009pe.ro/rezultate.html
[21] http://www.bec2009p.ro/rezultate.html
[22] http://www.infoelectoral.mir.es/min/
[23] http://www.bfs.admin.ch
[24] http://de.wikipedia.org/wiki/Kategorie:Gemeinde_in_Österreich
[25] http://www.mapy.cz
[26] Institut Géographique National, *Répertoire géographique des communes* http://professionnels.ign.fr/ficheProduitCMS.do?idDoc=5323862
[27] IGN GEOFLA, 2001, National Statistics
[28] VG250 Bundesamt für Kartographie und Geodäsie, 2007
[29] Local Councils, Ministero dell'Ambiente
[30] Powiaty W Polsce, GUS, Warszawa, 2003
[31] Instituto Nacional de Estadística
[32] http://fr.wikipedia.org/wiki/Liste_des_communes_de_Suisse
[33] http://www.u-cergy.fr/fr/laboratoires/labo-lptm/donnees-de-recherche.html
